# LFM: A Lightweight LCD Algorithm Based on Feature Matching between Similar Key Frames

**DOI:** 10.3390/s21134499

**Published:** 2021-06-30

**Authors:** Zuojun Zhu, Xiangrong Xu, Xuefei Liu, Yanglin Jiang

**Affiliations:** School of Mechanical Engineering, Anhui University of Technology, Ma’anshan 240302, China; mrzero1956@163.com (Z.Z.); liuxf618@163.com (X.L.); jiangyl96@163.com (Y.J.)

**Keywords:** SLAM, LCD, deep learning, object detection, feature matching, lightweight CNN, transformer

## Abstract

Loop Closure Detection (LCD) is an important technique to improve the accuracy of Simultaneous Localization and Mapping (SLAM). In this paper, we propose an LCD algorithm based on binary classification for feature matching between similar images with deep learning, which greatly improves the accuracy of LCD algorithm. Meanwhile, a novel lightweight convolutional neural network (CNN) is proposed and applied to the target detection task of key frames. On this basis, the key frames are binary classified according to their labels. Finally, similar frames are input into the improved lightweight feature matching network based on Transformer to judge whether the current position is loop closure. The experimental results show that, compared with the traditional method, LFM-LCD has higher accuracy and recall rate in the LCD task of indoor SLAM while ensuring the number of parameters and calculation amount. The research in this paper provides a new direction for LCD of robotic SLAM, which will be further improved with the development of deep learning.

## 1. Introduction

Nowadays, considerable research contributions have been made toward the evolution of visual Simultaneous Localization and Mapping (SLAM) [1,2,3]. SLAM plays an essential role in robot applications, intelligent cars, and unmanned aerial vehicles. As for robot applications, SLAM helps mobile robots solve two key problems: “Where am I?” and “How am I going?”. SLAM is also helpful for augmented reality and virtual reality applications [4]. Considering the high cost of laser SLAM, based on the laser range finder, and the limitations of its application scenarios, researchers have begun to focus on low-cost and information-rich visual SLAM in recent years. Among them, visual place recognition of pre-visited areas, widely known as Loop Closure Detection (LCD), constitutes one of the most important SLAM components [5]. Accurate closed-loop detection technology can eliminate pose drift and reduce cumulative errors to obtain globally consistent trajectories and maps. When working outdoors, GPS can be used to provide global location information [6]. However, indoors, we need to do something different.

Initially, the researchers used the similarity between images and maps to determine the consistency between image points and map points. However, this method is only suitable for the closed-loop detection of global maps in a small range of environments due to high computational complexity. Hahnel et al. proposed a method based on the odometer to determine whether the camera moved to a previous position by using the geometric relationship of the odometer [7]. This approach is ideal, but because of the existence of cumulative error, we cannot correctly know whether the robot moved to a certain position before.

The other method is based on appearance, which only carries out loop detection according to the similarity of two images. By detecting the similarity between the current frame image obtained by the robot and the historical key frame image, it can judge whether the robot has passed the current position. Appearance-based methods mainly include local LCD and global LCD. A system based on local appearance searches for the best image match to a set of adjacent images and uses similar comparison techniques [5]. Milford et al. proposed a sequence-based approach that calculates the best candidate matching positions in each local navigation sequence and then achieves localization by identifying the coherent sequences of these matches [8]. Another system based on global appearance finds the most similar images in the database in an exhaustive way. For instance, the Bag of Words (BoW) method proposed by Mur-Artal et al. is a global detection system [9]. In this method, a visual dictionary is constructed by clustering the data composed of the image features of the training set. Then, the image of the current frame is mapped to the visual dictionary and calculate the similarity of the feature description vector between the image of the current frame and the image of the historical frame. However, the limitation of this method is that the period of image feature extraction is several times that of other methods. However, this method is limited by the speed of image feature extraction, and the feature extraction cycle is several times longer than other steps. For low-texture features, Joan P. et al. proposed the LiPo-LCD method based on the combination of lines and points, which adopt the idea of incremental Bag-of-Binary-Words schemes, and build separate BoW models for each feature, and use them to retrieve previously seen images using a late fusion strategy [10].

In other words, an appearance-based LCD problem can be transformed into an image retrieval task. In this case, the current input image is treated as a query image, while the previous image is treated as a database image [11]. Therefore, the core factors affecting the performance of the appearance-based LCD method are image feature extraction and candidate frame selection. In most cases, existing methods work well. However, in some complex scenarios, such as changing lighting, dynamic object occlusion, and repetitive environments, these methods still face significant challenges. In this case, we need to consider the repeatability and discriminability of features. Secondly, the traditional BoW framework displaces the spatial information between visual words, resulting in quantization errors [12]. Sparse feature matching used to be solved with hand-crafted descriptors [13,14,15]. Recently, Convolutional Neural Networks (CNN) has had great success in pattern recognition and computer vision tasks [16,17]. Many researchers use the depth features extracted by CNN to improve the LCD algorithm and achieve fine results [12,18,19]. However, the calculation amount of LCD based on CNN is quite enormous.

**Figure 1:**
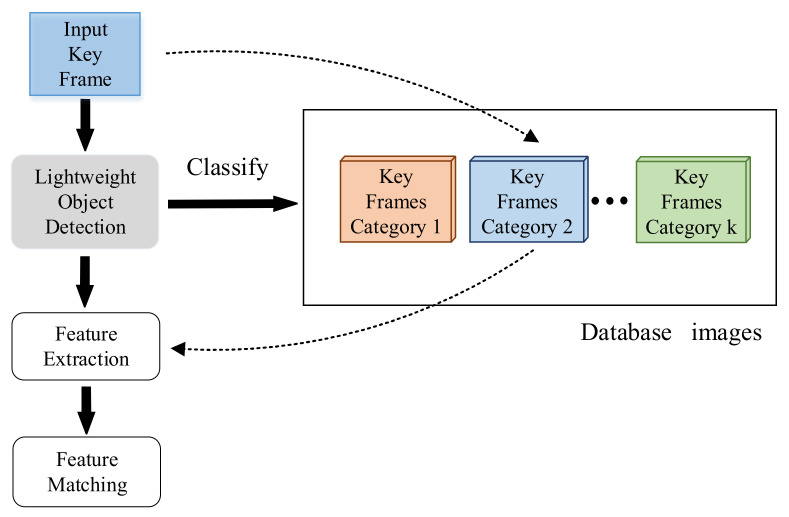
This is an overview of the proposed LFM framework; dashed lines indicate the category of the input key frame image. The database image consists of the key frames image entered previously.

To solve the above problems, this paper proposes LFM: a lightweight feature matching algorithm based on candidate similar frames. The framework (shown in Figure 1) includes the following steps:

1. Object detection of images form key frames based lightweight CNN;
2. Classify the images based on the salient features of the target detection results;
3. Classify the new input key frame images by a binary classified tree according to their labels, and then conduct lightweight feature matching for this image and the others from the same category, which could help the robots judge whether they have passed the current position before.

At the same time, considering the problem of computation and precision, we improve an ultra-lightweight CNN network to extract features. Experimental results show that LFM is especially superior in the LCD task. Summarized, our core contributions in this paper are:

- Based on the Residual Inverted Block proposed by MobileNetV2 [20], we designed a Residual Depth-wise Convolution Block (also called fish-scale block)to obtain more abundant depth information with less computation.
- We applied the new lightweight CNN (FishScaleNet) to the object detection algorithm, which has a high mAP value while improving the speed and creates a binary classified tree for the key images according to their labels.
- We applied the new lightweight CNN to the feature matching algorithm based on depth features, which can efficiently complete the matching task in the similar image categories. Compared with other feature matching algorithms, our lightweight feature matching algorithm has faster matching speed and less mismatching. Moreover, the LCD algorithm based on LFM can still guarantee better accuracy in the case of a high recall rate.

## 2. Related Works

### 2.1. Lightweight CNN

In recent years, many researchers have devoted themselves to optimizing the memory storage of convolutional neural networks and accelerating computation. The methods can be divided into the following directions: network pruning [21], low quantization [22,23], knowledge distillation [22], and compact network design [20,24,25,26]. The compact network design minimizes the loss of precision and reduces the total number of parameters and operations, so it is widely used. The commonly used compact method is to use group convolution to reduce the amount of computation, which can be reduced to 1/G (where G represents the number of groups). Particularly, when G is equal to the number of channels, this is called depth-wise convolution. Collet et al. proposed, for the first time, to use the combination of depth-wise (DW) convolution and pointwise (PW) convolution to obtain depth information [27]. The MobileNet series [20,28,29] proposed by Andrew et al. is the most successful lightweight CNN model based on depth-wise separable convolution so far. It emphasizes that the first PW convolution is responsible for channel spread. The second PW convolution is responsible for learning new features from different channels in the reverse residuals block, which greatly reduces the computational complexity. The GhostNet [25] proposed by Han et al. reduces the number of output channels of PW convolution and reduces the memory occupied by external features. In order to maintain the consistency of the output dimension, a series of linear transformations, such as DW convolution, are used to generate the internal features, which are fused with the output of PW convolution into the final feature vector. Yang et al. proposed an asymmetric bottleneck network based on MobileNet and adjusted the first PW convolution dimension. Moreover, the flow of information is enriched through feature reuse and the computational savings are migrated to the second point-by-point convolution [26].

### 2.2. Object Detection

Before deep learning, traditional object detection algorithms are mainly divided into two stages of feature extraction and classification, but the correlation between extracted features and classifier is quite weak. The RCNN [30] proposed by Girshick et al. is a pioneering CNN-based target detection algorithm network. Subsequently, the proposed Fast R-CNN replaces SVM classifier with SoftmaxLoss and adopts Region of Interest (ROI) pooling. These improvements combine classification and regression to improve the accuracy of the algorithm. Based on the Fast R-CNN, researchers then proposed Faster R-CNN [16] with Region Proposal Network (RPN): the suggestion box can be obtained by sliding to find the accurate position of the target object. Considering that the performance of the detector is often limited by the imbalance in the process of training, Pang et al. proposed LIBRA R-CNN. This is a balanced training method that includes IoU balanced sampling, balanced FPN and balanced L1 loss. However, in general, these two-stage methods are not perfect in terms of detection speed.

The YOLO series [31,32,33] proposed by Redmon et al. is a one-stage target detection method. It divides the image into grids and generates a category and two target boxes in each grid. Then the classification and regression of the boxes are unified into a loss function for learning. Because the entire detection pipeline is a single network, the detection performance can be directly optimized end-to-end, so it is also extremely fast. YOLO—different from sliding window and RPN—implicitly encodes contextual information during training, so that it can better understand the global information of pictures. Bochkovskiy et al. proposed YOLOv4 [34], a version with lots of improvement of Yolov3, which modifies state-of-the-art methods and makes them more efficient and suitable for single GPU training.

Recently, the Transformer network, which makes a lot of sense in Natural Language Processing (NLP), has been used in Computer Vision to great effect. Carion et al. proposed DETR [35]: an end-to-end Transformer target detection algorithm. The main component is a set based global loss function that enforces a unique prediction through binary matching and the Transformer encoder-decoder architecture. The DETR is the most efficient way to consider the relationship between the target object and the global image context, but it takes up too much of the GPU.

### 2.3. Feature Extraction and Matching

Feature points usually consist of key points and descriptors. Traditional feature matching methods, such as Lowe’s Scale-Invariant Feature Transform (SIFT) [14] algorithm, require a huge amount of computation and cannot be used for real-time feature matching. Then, Bay et al. improved SIFT and proposed the Speeded Up Robust Features (SURF) [13] algorithm, which uses detector metrics based on the Hessian matrix and descriptors based on distribution. Rublee et al. proposed a very fast binary descriptor based on BRIEF, called ORB [15], which is rotation invariant and resistant to noise.

However, most descriptors are still sensitive to large affine transformations, such as changes in scale and orientation. Consequently, modern feature matchers use multi-scale detection and orientation estimation [36].

In recent years, many researchers have proposed feature extraction and matching algorithms based on CNN. Tian et al. proposed a progressive sampling method, L2-Net [37], which uses CNN to learn high-performance descriptors in Euclidean space, and emphasizing the relative distance between descriptors for local patch matching. To learn consistent descriptors, Keller et al. proposed a novel mixed-context loss and scale-aware sampling method, which takes advantage of the scale consistency of Siamese Loss and the faster learning ability of Triplet Loss [38]. Cieslewski et al. proposed a method of matching points of interest without descriptor (different from the traditional work of matching points according to description) [36]. This network has multiple output channels and can implicitly match the corresponding points of two images.

Notably, LF Net [39] and SuperPoint [40] both consider traditional feature detection as separate units. The LF NET proposed by Yuki et al. is a network that can learn local features from scratch and is constrained in a self-monitoring manner, requiring only image sequences with the ground true depths and poses. LF NET uses single CNNs for multi-scale interest point detection, feature direction estimation and feature description. The difference is that the SuperPoint proposed by Daniel et al. consists of only one interest point detector and one network of feature descriptors, and the two networks share multiple encoder layers. The authors of SuperPoint then propose SuperGlue [41]: an attention-based graph neural network for local feature matching algorithms. It includes self-attention and cross-attention, which can simultaneously enhance the receptivity field of local descriptors and realize cross-image communication.

Recently, Vision Transformer (ViT) has been used more and more in the field of computer vision, such as object detection [35,42], image classification [43] and semantic segmentation [44]. Inspired by SuperGlue, Sun et al. proposed the LoFTR [45] algorithm. It is a local feature matching algorithm based on Transformer without the feature detector, which performs well in terms of speed and accuracy as well as in the case of repeated texture.

## 3. Methods

In this section, we introduce the framework of LFM-LCD in detail. These include: a lightweight CNN based on Fish Convolution Block; object detection based on YOLOv4 with our lightweight CNN instead of CSPDarkNet [46]; a classification tree with a structure that is similar to the BoW dictionary; an improved feature matching network of LOFTR [45].

### 3.1. FishScaleNet

Deep separable convolution decomposes the standard convolution process into two operations. The first operator, called depth-wise convolution, uses a single-channel filter to learn the spatial correlation between positions in each channel separately. The second operator is a 1 × 1 convolution, which is used to learn new features by computing linear combinations of all input channels.

Standard convolution takes an input tensor $L_{i}(h_{i}\times w_{i}\times c_{i})$, and applies convolutional kernel $K\in R^{k\times k\times c_{i}\times c_{j}}$ to produce an output tensor $L_{j}(h_{i}\times w_{i}\times c_{j})$. Standard convolutional layers have the computational cost: $h_{i}\times w_{i}\times c_{i}\times c_{j}\times k\times k$. Depth-wise separable convolutions are a drop-in replacement for standard convolutional layers. Empirically they work almost as well as regular convolutions, but only cost:$h_{i}\times w_{i}\times c_{i}\times (k^{2}+c_{j})$.

When the size of the convolution kernel is 3×3, the computational effort of the deep separable convolution will be reduced by nearly 9 times.

**Figure 2:**
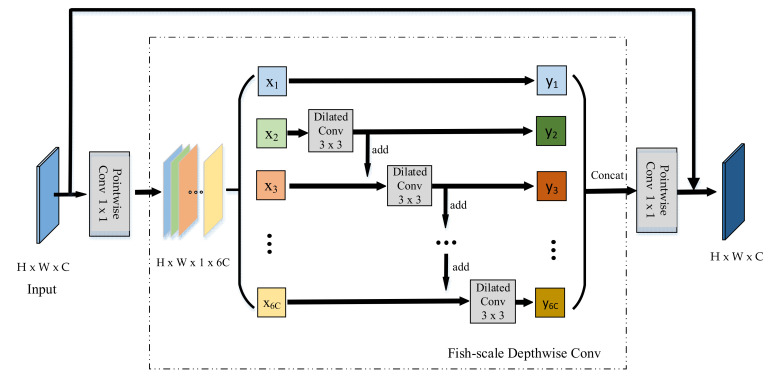
Skeleton of the fish-scale convolution block; H, W, and C denote the height, width, and channel dimension. The number of channels expand six times after point-wise convolution. After the depth dilated convolution, each layer feature is added into the next layer, and then dilated convolution of the next layer is conduct.

Inspired by MobileNet [20,28], a novel convolution block was proposed based on the inverted residual block, which depth-wise convolution is related to each other and transmitted in turn. Its structure is like a fish scale (it can be seen in Figure 2); therefore, we call it a fish-scale convolution block.

Fish-scale convolution works almost as well as inverted residual convolutions but costs just $h_{i}\times w_{i}\times (c_{j}-2)$ more than the latter: $h_{i}\times w_{i}\times [(c_{j}-2)+c_{i}\times (k^{2}+c_{j})]$.

Reference [47] proposed that the success of FPN is due to its divide-and-conquer solution to the object detection optimization problem, where dilated convolution plays an indispensable role. Dilated convolution mainly solves the problem of data structure loss in the space of standard convolution.

We adopted the Dilated Convolution [48], which is different from other convolution blocks of different lightweight CNNs; it increases the receptive field without sacrificing the size of the feature map. The receptive field of the dilated convolution can be calculated as:

(1) $$RF_{i}=RF_{i-1}+(K-1)\times s$$

(2) K=k+(k−1)(r−1)

where $RF_{i-1}$ represents the size of the receptive field at the upper layer, K denotes the size of the new dilated convolution kernel, and k denotes the size of the standard convolution kernel; s indicates the stride of the dilated convolution, and r denotes the dilated rate.

As for k = 3, r = 2i, it is easy to get that size of the receptive field of each element in $RF_{i+1}$ is $\left(2^{i+2}-1)\times (2^{i+2}-1\right)$; therefore, the receptive field is a square of an exponentially increasing size.

Based on the design of the fish-scale block structure, we further designed a lightweight CNN framework with strong practicability, named FishScaleNet. It follows the design rules of MolieNetv3, as shown in Table 1.

As shown in Table 2, we compared the computational complexity of MobileNetV1-v3 and FishScaleNet, and pointwise convolution accounted for most of the computational amount. However, FishScaleNet relatively increases the proportion of deep convolution and obtains richer spatial correlation between features. It is of great help to the downstream work (target detection and feature matching) of our LCD task, and the accurate results are obtained.

### 3.2. Lightweight YOLOv4

An ordinary object detector is composed of the backbone, neck, and head. The backbone network is used to extract the preliminary features, while the second part is used to extract the enhanced features. The final head is to get the predicted result. The backbone network of YOLOv4 is CSPDarknet [46], the neck is SPP [49] and PANet [50], and the head is Yolo Head [33].

**Figure 3:**
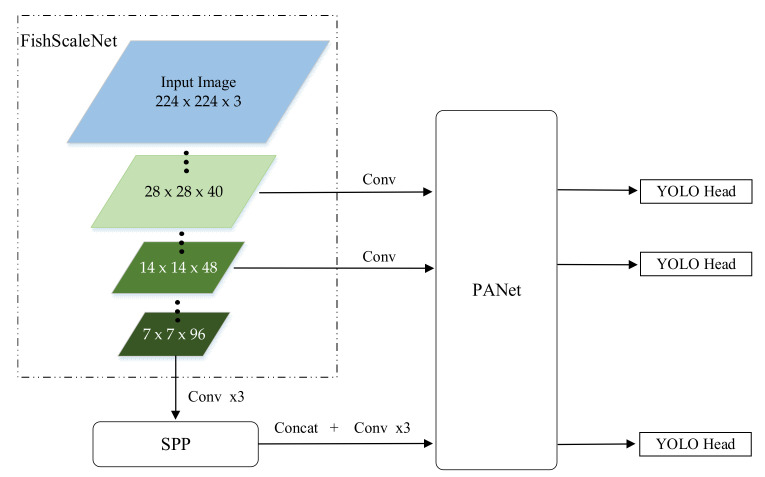
Pipeline of the lightweight YOLOv4. The size of the input image is 224 × 224 × 3, and three effective feature layers with different initial sizes are obtained through FishScaleNet, which are, respectively, introduced into the enhanced feature extraction network: SPP and PANet. The three preliminary effective features were fused to obtain a more effective feature layer, and input to the predictive convolution head, YOLO Head, to obtain the detection results.

As shown in Figure 3, we replaced its backbone, CSPDarkNet, with a lightweight FishScaleNet, and kept the other main structures, such as Bag of Freebies (BoF) and Bag of Specials (BoS) for detector: CIoU-loss, CmBN, DropBlock regularization, mosaic data augmentation, self-adversarial training, eliminate grid sensitivity, using multiple anchors for a single ground truth, cosine annealing scheduler, optimal hyper-parameters, random training shapes, Mish activation, SPP-block, SAM-block, PAN path-aggregation block, DIoU-NMS [34].

### 3.3. Binary Classified Tree

K-ary tree classifier is a fast graph classification algorithm, especially for large-scale graphs. The main idea of k-ary tree is to project the whole graph onto a set of optimized features in the common feature space without any prior knowledge of the subtree pattern. Then, a traversal table is constructed to track similar patterns in the optimization data [51].

**Figure 4:**
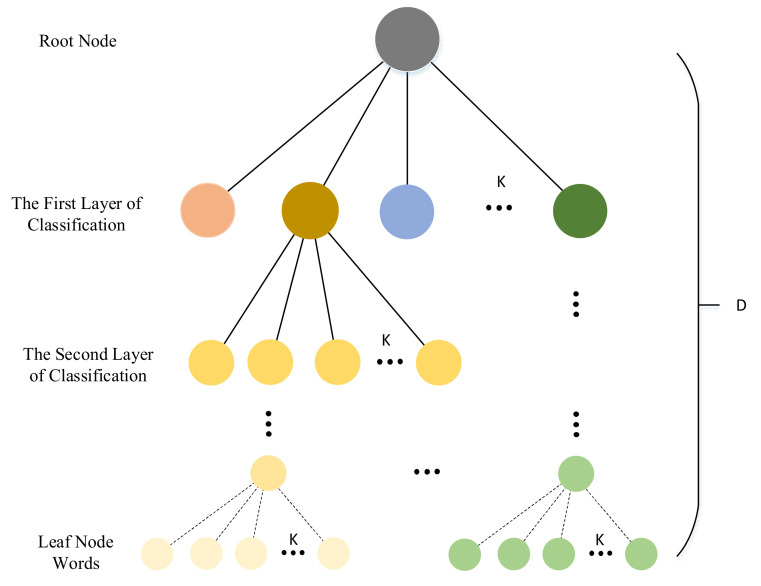
This is the structure of the k-ary tree of BoW. Different colors represent different clusters. The cluster number of each node is k, and d denotes the depth of the tree.

The k-ary tree in BoW [9] is a hierarchical K-means clustering, which obtains a tree with the depth of D and the branch of K, which can store KD words. It can be seen in Figure 4.

**Figure 5:**
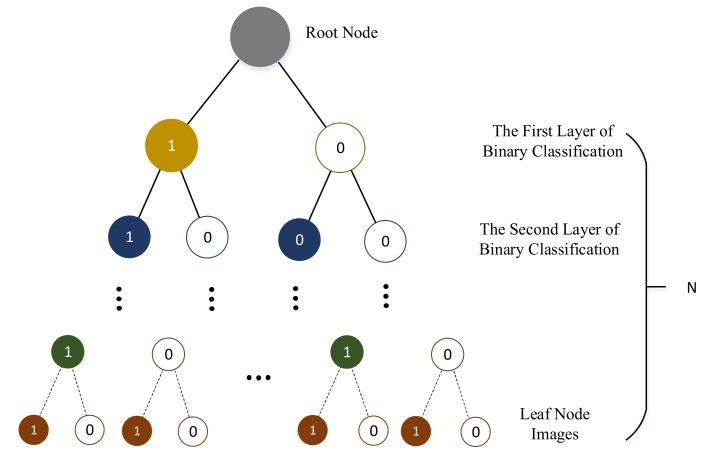
This is the structure of the Binary Classified Tree. Schemes follow the same formatting. Different colors represent the binary classification results of different labels; 1 indicates this label is present, 0 indicates this label is not present. The depth of the tree is the total number N of categories of object detection.

In LFM-LCD, we can directly use the result of object detection in the previous step. Sort the labels of the class detected by the target by the actual space size. Then the image is classified according to its detection label (predictions with rejection probability less than 0.5) in this order, and the binary sequence of each image is obtained. The classification results are represented by vectors. The structure can be seen in Figure 5.

(3) $$c(i)=\left\{\begin{array}{ll} 1, & if\;l_{i\;}\;exist\\ 0, & otherwise \end{array}\right.\;i\;=\;1,2,3,\ldots ,N$$

(4) c=( c(1) , c(2) , … ,c(N) )

where **c** denotes the vector quantity of the binary classification. The $l_{i\;}$ represents the category labels.

We eliminated the labels with a predicted probability of less than 0.5, and we also had to remove the non-influential labels in this scenario to expand the classification. We randomly extracted the classification vectors of M images,$c_{1},c_{2},\ldots ,c_{M}$. Then, the number of repeated appearances of each label in the M pictures, p(i) was calculated. Finally, we can calculate the influence E(i)=p(i)/M of the label and remove the labels whose E(i) was less than the threshold value.

(5) $$p(i)\;=\;\sum_{j=1}^{M}c_{j}(l_{i})\;$$

We can get the category of each key frame through the binary classification tree. Then, we can carry out feature matching between images of the same category. In order to eliminate the error caused by the different detection results of the target detection algorithm for small objects, the order of binary classification is carried out from large objects to small objects. Set the number of images of the same category for feature matching to be at least m. When the current frame image enters the classification tree, if there is no image of the same category or the number is less than m, the upper binary classification can be traced to improve the accuracy of LCD.

### 3.4. Feature Matching Based on LoFTR

**Figure 6:**
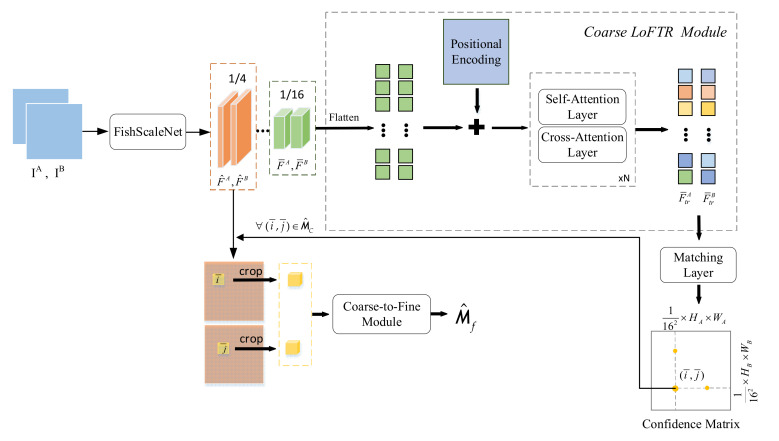
This is the overview of the lightweight LoFTR for feature matching. I^A^ and I^B^ denotes the input image. ${\bar{F}}^{A}$, ${\bar{F}}^{B}$, ${\hat{F}}^{A}$ and ${\hat{F}}^{B}$ are the feature map extracted by FishScaleNet. Coarse LoFTR module is based on vision Transformer. $M_{c}$ represents the confidence matrix between features of I^A^ and I^B^. ${\hat{M}}_{c}$ indicates the $M_{c}$ after removing the outliers. ${\hat{M}}_{F}$ denotes the fine-level matching.

Compared with other feature matching algorithms, such as ORB [15] and SuperPoint [40], LoFTR [45] can solve the repeatability problem of feature detector. This is critical for the LCD task in SLAM. It is similar to LOFTR, i.e., our feature matching task performed fine-level feature matching after coarse-level feature matching. The difference is that we did not use standard CNN to extract features. Instead, we used FishScaleNet proposed in Section 3.1. Meanwhile, we chose a feature map with a smaller size to further reduce the calculation amount of feature matching. The overview of the lightweight LoFTR can be seen in Figure 6.

#### 3.4.1. Lightweight Feature Extraction

For two similar images I^A^ and I^B^ in the same category, we used FishScaleNet to extract the multi-level features of the two images. LoFTR uses features extracted at 1/8 and 1/2. For lightweight FishScaleNet, ${\bar{F}}^{A}$ and ${\bar{F}}^{B}$ are used to represent coarse features extracted at 1/16 of the original image size, ${\hat{F}}^{A}$ and ${\hat{F}}^{B}$ are used to represent fine features extracted at 1/4 of the original image size.

#### 3.4.2. Coarse LoFTR Module

LOFTR converts ${\bar{F}}^{A}$ and ${\bar{F}}^{B}$ into easier features, represented by ${\bar{T}}_{F}^{A}$ and ${\bar{T}}_{F}^{B}$. In the self-attention layer, the input vectors are Q (query), K (key), and V(value). The query vector Q obtains attention weight information according to its dot product with the K vector and the corresponding with V. It is expressed as follows:

(6) $$A\mathrm{ttention}(Q,K,V)\;=\;softmax\;(QK^{T})V$$

We used the same position encoding approach as DETR [34]. In the self-attention layer, the two groups of the encoded features are the same, while in the cross-attention layer, the cross direction determines the change of features. After the self-attentional layer and the cross-attentional layer, we get two new groups of features ${\bar{T}}_{F}^{A}$ and ${\bar{T}}_{F}^{B}$. The scoring matrix between the new features is S:

(7) $$S\;(i,j)=\;\frac{1}{\Gamma }\;\cdot \;\langle {\bar{T}}_{}^{A}(i)\;,\;{\bar{T}}_{}^{B}(j)\rangle$$

After the new feature passes through the matching layer, the confidence matrix $M_{c}$ of the coarse level feature is obtained, and its size is $\frac{H_{A}W_{A}}{16^{2}}\times \frac{H_{B}W_{B}}{16^{2}}$. Where, the matching probability in $M_{c}$ is obtained by softmax on both dimension:

(8) $$M_{C}(i,j)=\;softmax{\left(S(i\;,\;\cdot \;)\right)}_{j}\;\cdot \;softmax{\left(S(\;\cdot \;,j)\right)}_{i}$$

Then Mutual Nearest Neighbor (MNN) is used to eliminate outliers in the confidence matrix to obtain ${\hat{M}}_{c}$.

(9) $${\hat{M}}_{C}=\left\{\left(\bar{i},\bar{j})\;|\;\forall \;(\bar{i},\bar{j})\;\in \;MNN(M_{C}\right)\right\}$$

#### 3.4.3. Coarse-to-Fine Module

For arbitrary coarse matching $(\bar{i},\bar{j})\in {\hat{M}}_{C}$, it is projected into 1/4 fine feature to obtain a small matching window. Since the proportion of coarse-to-fine is 4:1, the cropped feature $\hat{F}\in {\mathbb{R}}^{4\times 4}$. Two local fine features A and B are generated through LOFTR. By calculating the expected value of the probability distribution, the subpixel accuracy of the final position $\hat{j}{}^{\prime }$ is obtained. All marches are collect $\left\{(\;\hat{i},\hat{j}{}^{\prime })\right\}$ to generate the final fine-level match ${\hat{M}}_{F}$.

## 4. Experiments

### 4.1. Experimental Setup

**Figure 7:**
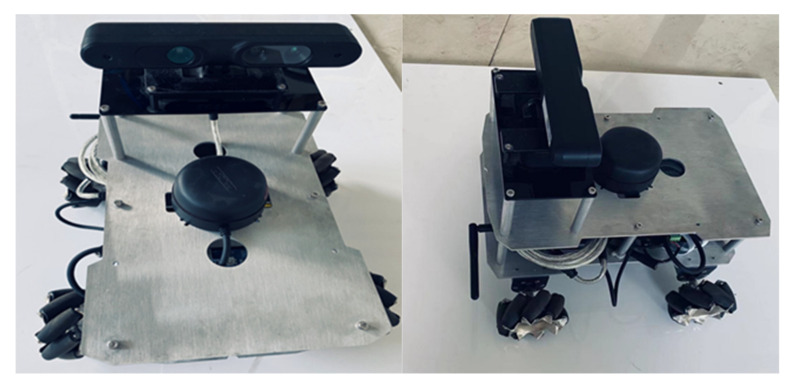
The vehicle with the RGB-D camera.

This work uses an RGB-D camera as the vision sensor to carry out the SLAM experiment on a mobile vehicle. As shown in Figure 7, the camera type is “LETV Pro Xtion”. The specific parameters are shown in Table 3.

We utilized the deep learning framework PyTorch to implement the FishScaleNet models for object detection and feature matching. We used the standard SGD optimizer for training, with the momentum and weight decay, respectively, set at 0.9 and 0.0005. We used the step decay learning rate scheduling strategy with the initial learning rate 0.01. All architecture used a single GPU to execute multi-scale training in the batch size of 64. Other experiments used default settings.

### 4.2. Object Detection for Key Frames

In order to verify the practicability and accuracy of FishScaleNet in LFM-LCD, since the lightweight network focuses on ensuring the necessary accuracy while reducing the numerous parameters and calculation, we only compare the image classification performance of FishScaleNet with other lightweight networks (MobileNetV2 [20] and GhostNet [25]) on ImageNet and the experimental results of object detection on COCO dataset. The one-stage YOLOv4 [34] and the two-stage Faster RCNN [16] were tested for the object detection framework. The result of performance on ImageNet is shown in Table 4, and the test result of object detection on COCO datasets can be seen in Table 5.

In the image classification task of ImageNet, our FishScaleNet achieved Top1 accuracy, which is similar with MobileNet and GhostNet in the minimum number of parameters and computation. We can finish the task faster on the same computer.

**Figure 8:**
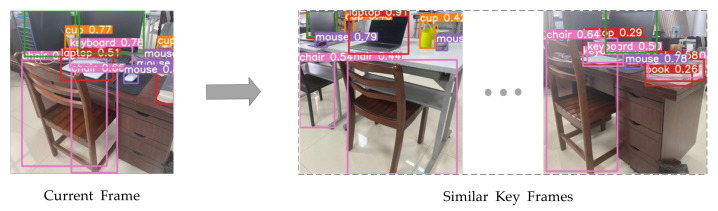
Object detection for the current frame and finding similar key frames by the binary classified tree.

For target detection on COCO dataset—Faster RCNN in two-stage and YOLOv4 in one-stage were our target detection frameworks. Experiments show that our FishScaleNet can achieve higher mAP value and faster speed on the YOLOv4 framework. At the same time, our lightweight target detection network has a certain feasibility in the LCD task. The current frame image was transmitted to the network for the object detection task, and object labels (as shown in Figure 8); cups (0.77), chairs (0.66), laptops (0.51), and books (0.34) were obtained. Labels with predictive probability less than 0.5 (such as books) were eliminated, and then binary classification was carried out on the images. Finally, the binary vector results of classification were passed into the key frame database of the same category for the feature matching task.

Of course, in similar environments, objects of the same kind happen to be present in the images, such as the different tables in our lab (Figure 8), and are classified into the same category. However, it did not affect the LCD task of our robot in SLAM because the feature matching task will continue to help us eliminate these possibilities.

### 4.3. Feature Matching between Similar Key Frames

**Figure 9:**
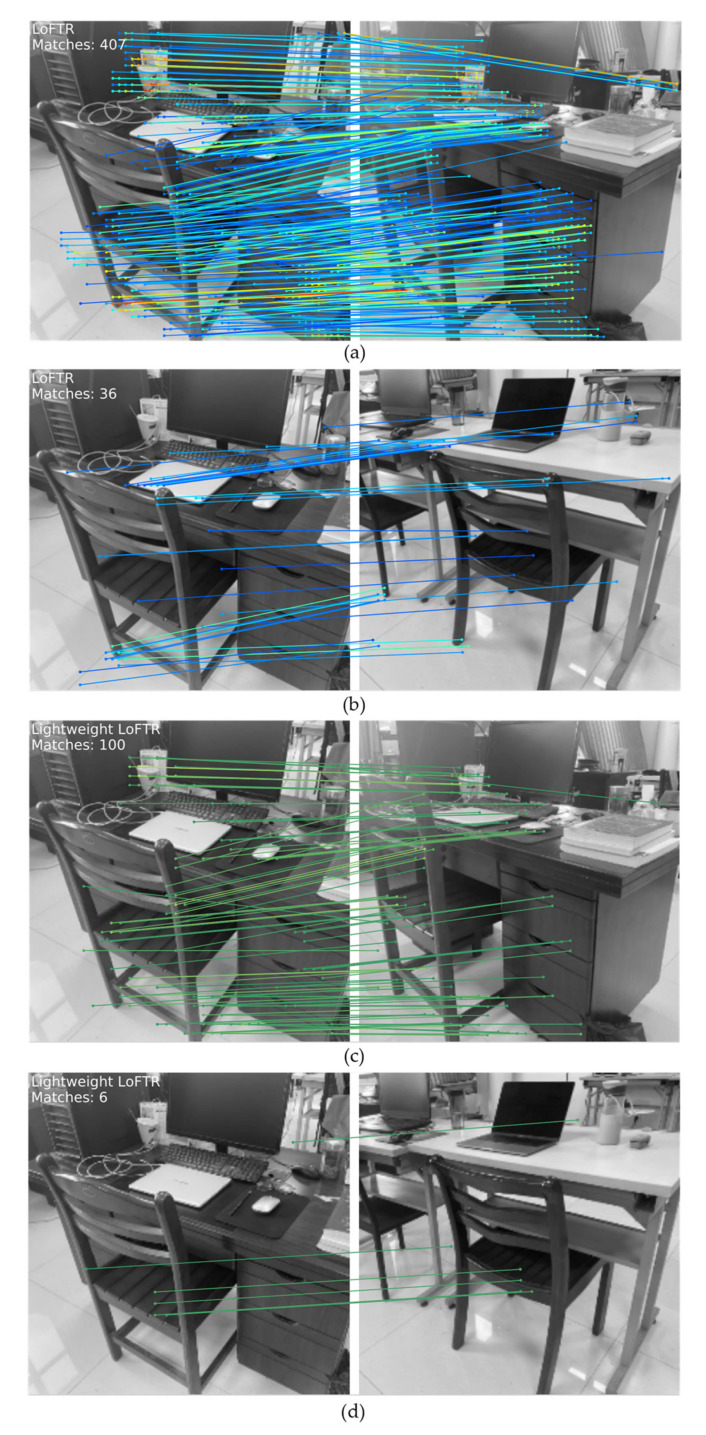
Feature matching between the current frame and similar images in the database. (**a**) Loop closure frames with standard LoFTR. (**b**) Non-loop closure frames with standard LoFTR. (**c**) Loop closure frames with lightweight LoFTR. (**d**) Non-loop closure frames with standard LoFTR.

A mass of experimental data in [45] demonstrates the accuracy and superiority of LOFTR for feature matching in low-texture regions as well as symmetric and repeated regions, and the performance of relative pose estimation and visual positioning on multiple data sets reaches the most advanced level. Here, we only compare the feature matching results of the lightweight LOFTR with the standard LoFTR. As shown in Figure 9a, standard LoFTR will notice a large amount of deep feature data, and abundant calculations are not suitable for our real-time LCD task. For non-loop-closure frames, there will also be nearly 9% false matches, as shown in Figure 9b. For our lightweight LOFTR, as shown in Figure 9c, feature matching can be performed quickly for only 1/4 calculations of the former, providing real-time data support for our LCD. In addition, for non-loop-closure frames, as shown in Figure 9d, our mismatching rate is about 6%, which improves the accuracy and recall rate of LCD tasks. In summary, the lightweight LOFTR can quickly identify the relationship between the current frame and similar key frames, which greatly helps the robot to determine whether the current position is loop or not.

### 4.4. Comparative Experiments of LCD

The proposed LFM-LCD algorithm was evaluated on indoor and outdoor open datasets, respectively, as shown in Table 6. It is compared with other LCD methods proposed recently, such as DOSeqSLAM [5], DXSLAM [10], and LiPo-LCD [52].

#### 4.4.1. Datasets

Given the complexity of the LFM-LCD algorithm, real-time results may not be achieved for high FPS inputs. Therefore, we verified the feasibility of our LFM-LCD algorithm on the KITTI 05 and the indoor ICL-NUIM, respectively; the datasets were obtained by the hand-held sensor. Therefore, we can set the input of the key frame as less per second.

#### 4.4.2. Comparative Results

The ground truth (GT) is the information data of the actual loop closure event that occurrs in the dataset. GT is structured as a binary matrix with image-to-image correspondences, where the ones ($GT_{ij}=1$) denote the existence of a loop closure event.

It can be seen from Table 7 that, compared to other methods, LFM-LCD has a higher precision and recall rate in indoor scenes with the ICL-NUIM dataset. Although DOSeqSLAM has a 100% precision rate, its recall rate is only around 50%. However, in outdoor scenes, the precision and recall rate of LFM-LCD decreased.

**Figure 10:**
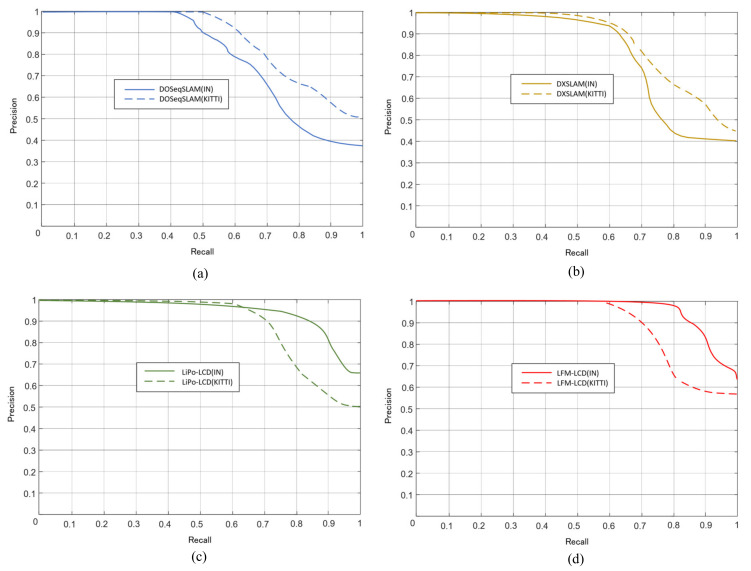
The RP-Curves of different LCD methods in different datasets. IN denotes the ICL-NUIM datasets and KITTI denotes the KITTI 05 datasets; (**a**) shows the RP-Curves of DOSeqSLAM; (**b**) shows the RP-Curves of DXSLAM; (**c**) shows the RP-Curves of LiPo-LCD; (**d**) shows the RP-Curves of LFM-LCD.

Precision = TP/(TP + FP), Recall = TP/(TP + FN). In this equation, true positive (TP) means loop closure for both GT and detected results. False negative (FN) means that GT is loop closure, but detection is not. A false positive (FP) indicates that the detected result is loop closure, but in fact it is not. We used Precision–Recall Metrics to evaluate our system. Precision is defined as the ratio between true-positive identifications and the total detections of algorithm. Recall is defined by the number of detected loop closure events over the actual events appearing in the ground true. The RP-Curve can be seen in Figure 10.

## 5. Discussions

As with the RP-Curve shown in Figure 10, compared with other methods, LFM-LCD has higher reliability in regards to indoor data sets of specific objects, which is quite conducive to SLAM of indoor robots. However, when we attempt to increase the key frame input frequency, our approach may not be able to achieve real-time results. Therefore, we still have a lot of room for improvement in speed and application for outdoor scenes needs to be improved too.

In addition, the area where the object features are not obvious will lead to a single classification, which will increase the burden of LFM. Therefore, we need to further optimize the label results of target detection in the future to solve this problem, e.g., by using semantic segmentation or instance segmentation instead of simple target detection networks. Of course, there are other problems, such as the complex scaling of the fish-scale block, which makes it less efficient. We still have a lot of work to do in the future, such as redesigning a more efficient lightweight backbone, or simplifying our methods to adapt to more real-time scenarios as much as possible.

## 6. Conclusions

This paper describes a deep-learning based SLAM loop detection algorithm. In view of the problems caused by cumulative errors in visual SLAM and the inaccuracy of existing loop detection algorithms, a new direction was proposed. Simple, divided into three steps: object detection for key frames, binary classification, feature matching. In consideration of the large number of parameters and computation amounts generated when the commonly used backbone extracted the preliminary features, we proposed another deep separable lightweight convolutional neural network inspired by MobileNetV2. The high receptive field was obtained by dilated convolution combined with the self-attention mechanism of Transformer, which can better complete the feature matching task between similar key frames. We used binary classification to obtain the classification vector of the key frame and add the dynamic weight of the label according to the category proportion in the binary classification tree. Compared with several advanced LCD algorithms, our proposed LFM-LCD has high advantages for low-speed SLAM for robots in indoor scenes.

## Figures and Tables

**Table 1:** Specification for FishScaleNet using MobileNetV3 base.

Input	Operator	t	c	s	r	n
224 × 224 × 3	Conv2d	-	16	2	-	1
112 × 112 × 16	Fish-Scale-Block	1	24	2	2	2
56 × 56 × 24	Fish-Scale-Block	6	40	2	4	2
28 × 28 × 40	Fish-Scale-Block	6	48	2	2	3
14 × 14 × 48	Fish-Scale-Block	6	96	1	2	4
14 × 14 × 96	Fish-Scale-Block	6	96	2	2	2
7 × 7 × 96	Fish-Scale-Block	6	96	1	1	2
7 × 7 × 96	conv2d 1 × 1	-	576	1	-	1
7 × 7 × 576	avgpool 7 × 7	-	-	1	-	1
1 × 1 × 576	conv2d 1 × 1	-	1024	1	-	1
1 × 1 × 1024	conv2d 1 × 1	-	1000	1	-	1

Each row shows a conv2d layer or a fish-scale block, repeated n times. t denotes the expansion factor in first pointwise convolution. s indicates the stride number of the convolution layer, and r denotes the dilation rate in the depth-wise convolution. c denotes the output channel size, and s denotes the stride number of the convolution layer. “Input” and “Operator” indicate the shape of the input tensor and the operator type.

**Table 2:** Computational complexity distribution of MobileNetV1-V3 and FishScaleNet.

Network	Depth-Wise	Point-Wise	Others
MobileNetV1	3.1%	95%	1.9%
MobileNetV2	6.2%	84.4%	9.4%
MobileNetV3	8.9%	88.5%	2.6%
FishScaleNet	16.6%	81.1%	2.3%

**Table 3:** The specific parameters of the “LETV Pro Xtion” camera.

No.	Information	Descriptions
1	Max Power Dissipation	2.5 W
2	Working Distance	0.8 m–3.5 m
3	Field of View	58 H,45 V,70 D
4	Sensor	Depth Sensor
5	Depth Image Size	VGA(640 × 480):30 FPS

**Table 4:** Performance on ImageNet of different lightweight CNN.

Network	Top 1-Acc	Parameters	MAdds	CPU
MobileNetV2 [20]	70.4%	3.4 M	300 M	92 ms
GhostNet [25]	**71.2%**	5.6 M	164 M	89 ms
FishScaleNet	70.6%	**2.9 M**	**157 M**	**86 ms**

**Table 5:** Results on MS COCO dataset.

Backbone	Detector	mAP	CPU
MobileNetV2 [20]	Faster RCNN [16]	26.4%	213 ms
GhostNet [25]		**27.4%**	187 ms
FishScaleNet		26.7%	**178 ms**
MobileNetV2 [20]	YOLOv4 [34]	27.1%	197 ms
GhostNet [25]		26.8%	172 ms
FishScaleNet		**27.3%**	**166 ms**

**Table 6:** Description of datasets.

Dataset	Description	Input of Key Frame/s
KITTI 05	Outdoor, dynamic, 10 Hz	1
ICL-NUIM	Indoor, static, 30 Hz	1

**Table 7:** Comparative Results.

Methods	Datasets	True Positives	False Positive	GT	Precision	Recall
DOSeqSLAM	KITTI 05	188	0	379	100%	50%
DXSLAM	KITTI 05	197	5	379	97.5%	53%
LiPo-LCD	KITTI 05	226	8	379	97%	61%
LFM-LCD	KITTI 05	236	13	379	95%	64.5%
DOSeqSLAM	ICL-NUIM	72	0	176	100%	41%
DXSLAM	ICL-NUIM	102	7	176	94%	60%
LiPo-LCD	ICL-NUIM	127	5	176	96%	74%
LFM-LCD	ICL-NUIM	143	2	176	99%	82%

## Data Availability

Publicly available datasets were analyzed in this study. Those data can be found here: [https://image-net.org/] (accessed on 30 June 2021) [https://cocodataset.org/] (accessed on 30 June 2021) [http://www.cvlibs.net/datasets/kitti/raw_data.php] (accessed on 30 June 2021) [https://www.doc.ic.ac.uk/~ahanda/VaFRIC/iclnuim.html] (accessed on 30 June 2021).
